# Mucocutaneous Involvement in Behçet's Disease: How Systemic Treatment Has Changed in the Last Decades and Future Perspectives

**DOI:** 10.1155/2015/451675

**Published:** 2015-06-22

**Authors:** Cinzia Rotondo, Giuseppe Lopalco, Florenzo Iannone, Antonio Vitale, Rosaria Talarico, Mauro Galeazzi, Giovanni Lapadula, Luca Cantarini

**Affiliations:** ^1^Interdisciplinary Department of Medicine, University of Bari, Piazza Giulio Cesare 11, 70124 Bari, Italy; ^2^Research Center of Systemic Autoimmune and Autoinflammatory Diseases, University of Siena, Viale Bracci 1, 53100 Siena, Italy; ^3^Rheumatology Unit, Department of Clinical and Experimental Medicine, University of Pisa, Via Roma 67, 56126 Pisa, Italy

## Abstract

Behçet's disease (BD) is a multisystemic disorder of unknown etiology characterized by the “triple symptom complex” consisting of recurrent oral aphthosis, genital ulcers, and chronic relapsing bilateral uveitis. Recurrent mucocutaneous lesions are generally considered the hallmark of the disease, being the most common symptoms presenting at the onset of disease. Although the improvement of knowledge about the pathogenetic mechanism added important changes in the treatment management of BD clinical manifestations, thus avoiding the appearance of serious life-threatening complications which are disease related, the mucocutaneous lesions are still the most nagging clinical manifestations to be treated. In this work we reviewed the current state of knowledge regarding the therapeutic approaches for mucocutaneous lesions of BD mainly based on controlled studies to provide a rational framework for selecting the appropriate therapy for treating these troublesome features of the disease.

## 1. Introduction

Behçet's disease (BD) is an inflammatory disorder of undetermined aetiology, which is recently and unanimously recognized as both autoimmune and autoinflammatory disease [1]. Indeed, many of its classical manifestations and the characteristics of the recurrent course overlap with those of monogenic autoinflammatory disorders [2–5].

It is hypothesized that the main pathogenetic elements are represented by genetic predisposition, mainly HLA dependent, and environmental factors. Furthermore, it is believed that a misdirected immune response, triggered by some microbial agents (as herpes simplex virus-1 and* Streptococcus sanguinis*), could play a pivotal role in the pathogenesis of BD [6]. In this regard, the abnormal activation of either innate and adaptive immunity with consequent interaction of both T lymphocytes (mainly Th1 and Th17 phenotype) and activated neutrophils would seem to be involved in the disease onset [7–9]. Many cytokines may contribute to the pathological mechanism of BD [6, 10–12]; high sera title of tumor necrosis factor- (TNF-) alpha is found in patients with active BD [13] and the role of TNF-alpha inhibition in the pathogenesis of ocular inflammation was described in mice models [14]. Interleukin- (IL-) 6 has been demonstrated to be related to BD activity and central nervous system involvement, as confirmed by its high levels in CSF [15]. Recent studies have suggested a role of IL-1 in BD; actually IL-1 high title is found in sera [16] and synovial fluid of BD patients [17]. Indeed IL-1 may play a key role in the pathogenesis of ocular [18] and mucocutaneous involvement [19], although in the latter case evidence from literature is not entirely encouraging [20–23].

BD is clinically characterised by multiple organ involvement, in particular by the “triple symptom complex,” consisting of recurrent oral aphthosis, genital ulcers, and recurrent bilateral uveitis. Besides this classical clinical trial, BD recognizes also other organ involvements, as summarised in Table 1 [24–28].

Mucocutaneous lesions are the earliest and the most frequent manifestations of BD which may anticipate by many years other typical clinical symptoms. The most common mucocutaneous lesions are oral aphthae (OA), which are included in the BD classification criteria. OA are characterized by recurrent and painful oral mucosa ulcerations. They manifest themselves, more frequently as minor aphthous ulcers (<10 mm in diameter) or, less frequently, as major ulcers (>10 mm in diameter, deeper and more painful than minor ulcers) or also as herpetiform ulcers (numerous, shallow, and small-pinpoint ulcers occurring in coalescing clusters). The genital ulcers (GU) are the second main symptom reported in the literature. They are similar in appearance and course to OA. The most frequently involved body areas are the scrotum in males and the major and minor labia in females. Cutaneous lesions, important characteristics of the disease, have been described as a major criterion for the classification. They mainly include erythema nodosum-like lesions, papulopustular lesions (sterile folliculitis-like lesions on an erythematous base), superficial thrombophlebitis, extragenital ulceration, and other cutaneous vasculitic lesions [29]. Skin pathergy reaction represents the unifying feature of the typical BD inflammation and is characterised by the presence of an abnormal skin reaction to traumatic insults or different types of inflammatory stimuli [30]. Moreover, pathergy phenomenon has no association with specific organ involvement or disease activity and is not only restricted to the skin [31].

The mucocutaneous manifestations are characterized by recurrent relapses; they usually have moderate to long-term course and their spontaneous resolution is rarely described. A wide number of conventional immunosuppressive drugs could be used to treat these lesions, but several failures, with lesion relapses, are commonly reported. The concomitant BD manifestations often drive the therapy management [32].

Herewith, we provide a review of the literature published on treatment strategies for mucocutaneous BD involvement, focusing on how treatment has changed in the last decades and on possible future perspectives.

*Hints from Treatment Guidelines*. In 2008 the EULAR treatment recommendations were published; they suggested treating skin and mucosa involvement both depending on the perceived severity and shared doctor-patient decision and according to dominant or concomitant manifestations. Topical measures (i.e., local corticosteroids) should be the first-line treatment for isolated oral and genital ulcers and acne-like lesions. Colchicine should be preferred when the dominant lesion is erythema nodosum. Azathioprine, INF-alpha, and TNF-alpha antagonist may be considered in resistant cases [32]. Some immunomodulator drugs, as tocilizumab and mycophenolate mofetil, have failed to reach a clinical improvement in the mucocutaneous lesions [28]. New drugs as apremilast seem to be effective in the treatment of oral and genital aphthosis [33]. Further studies may be necessary to prove the drug efficacy. The improvement of knowledge about the pathogenetic mechanism could add important changes in the treatment management of the BD clinical manifestation.

## 2. Literature Review

An electronic literature search was conducted using SCHOLAR, SCOPUS, and PUBMED. Case reports, open and double-blind trials, and cohort studies published up until 2015 were evaluated (Table 2). With regard to the drugs under clinical experimentation, the “clinicaltrials.gov” web site was consulted.

### 2.1. Past Decades

A wide range of drugs is available for treating mucocutaneous lesions in BD. In the past decades, traditional immunosuppressive agents were largely used to reduce the ulcers disability, but some contrasting results were observed, particularly in terms of maintaining remission effectiveness.

#### 2.1.1. Azathioprine

Azathioprine seems to be effective in controlling the progression of BD, especially in most critical manifestations, such as eye diseases. IARCT described a favourable effect on mucocutaneous lesions, as proven by statistically significant reductions in the frequency of oral and genital ulcerations; in particular, preventive effects were observed for GU and a healing improvement for OA [34].

#### 2.1.2. Colchicine and Antibiotics

It is well known that colchicine is recommended as a first-line therapy in erythema nodosum-like lesions in BD [32]. The effects on erythema nodosum were described in three double-blind, placebo-controlled trials [35–37]. In a double-blind trial the colchicine effects on reducing of GU were proven. No effects on OA and folliculitis were observed (the dose of colchicine was adjusted according to body weight) [36]. On the contrary, in another double-blind controlled crossover trial, OA, GU, and pseudofolliculitis improvements were described [37]. Two prospective studies, evaluating the association of colchicine and benzathine penicillin, have described the decrease in the frequency and duration of OA and erythema nodosum and an improvement of the frequency of GU [38, 39]. The beneficial effects of antibacterial therapy may be supported by the hypothesized role of streptococci in BD [1].

#### 2.1.3. Azithromycin

Azithromycin is a macrolide antibiotic characterized by wide spectrum of action. Its immunomodulatory effects are supposed [40]. The use of azithromycin in BD is based on previous hypothesis that* Streptococcus sanguinis* play a main role in pathogenesis of BD [41]. Two case series [42, 43] described the effectiveness of azithromycin in decreasing folliculitis and in fastening the healing time of oral ulcers.

#### 2.1.4. Minocycline

Minocycline is described to decrease the frequency of the OA, erythema nodosum lesions, and papulopustular lesions in an open study [44].

#### 2.1.5. Thalidomide

Despite failing in the treatment of eye involvement, one RCT [45], a pilot study [46], and three open studies [47–49] have demonstrated thalidomide effectiveness in the treatment of OA, GU, and papulopustular lesions, while an increase in the frequency of nodular lesion was reported. However, it is well known that thalidomide is associated with severe adverse events and birth defects, whereby its use is limited.

#### 2.1.6. Cyclosporine

Cyclosporine, a synthesis and release inhibitor of the IL-1 and IL-2, is frequently used in the treatment of eye involvements in BD, but just few evidences are described about mucocutaneous lesions. A double-blind trial [50], a controlled study [51], and an open study [52] showed that cyclosporine is effective in the GU and dermal lesion. The administrated dose is ranged from 5 to 10 mg/kg/day.

#### 2.1.7. Dapsone

Dapsone has proven to inhibit the chemotaxis of neutrophils [53]. A dose of 100 mg per day has been demonstrated to be very effective in healing the mucocutaneous lesions; as evidenced in an open study [54] and in a double-blind placebo-controlled clinical trial, dapsone showed relevant effects by decreasing the frequency and the duration of OA and the number and the frequency of GU [55]. No data are available regarding the duration of remission over time.

#### 2.1.8. Rebamipide

Rebamipide, a well-known gastric mucoprotective agent, used to treat gastritis and gastric ulcer in Japan, is observed to improve the aphthae count and to relieve the pain secondary to oral ulcers in a double placebo-controlled study. The authors conclude that it could be recommended as a long-term treatment for recurrent OA, also in association with other indicated drugs [56].

#### 2.1.9. Interferon-Alpha

Interferon-alpha, a large family of glycoproteins, seems to provide a cellular response to the foreign constituents of microbes, tumor, and antigens and is used in the treatment of several diseases. Although its mechanism of action is not well defined, INF-alpha seems to restore the low natural killer cell activity in patients with BD to a near normal level. Interferon alpha 2a, in BD has proven to reduce the duration and the pain of OA and the frequency of GU and papulopustular lesion, as well as erythema nodosum-like lesion, compared to placebo in a randomized placebo-controlled and double-blind study [57]. Similar results were found in 7 open studies [58–64]. In two previous reviews, the author highlighted that a treatment period of 2 to 4 months is at least necessary to obtain the highest effectiveness and declared a trend to disease relapse immediately or up to 7 months after treatment discontinuation [65] and rapidly responded after reinstitution of INF-alpha treatment [66]. An initially high dose (9 million units 3 times per week) for 3 months and then a low maintenance dose (3 million units 3 times per week) are recommended [53].

#### 2.1.10. Anti-CD 52

Alemtuzumab is a humanized immunoglobulin G1 monoclonal antibody that targets CD 52, a glicosilate antigen that is present on lymphocytes and macrophages. Its main effect is T-cell depletion. In a case report, an 18-year-old woman with polyarthralgias, orogenital aphthosis, and erythema nodosum resisted conventional immunosuppressive therapy, describing a long lasting remission after the sixth subcutaneous administration [67]. In an open trial, 18 patients with orogenital ulcerations, ocular involvement, and neurological involvement, treated with Alemtuzumab (134 mg), in 6 months in 72% reached a complete remission and the daily dose of prednisolone was reduced; therapy was discontinued in 33% for stable remission. The hypothyroidism development was the most common adverse effect recorded [68].

### 2.2. Current Treatment Option

In the last decade, the good efficacy of biotechnological drugs in the treatment of resistant mucocutaneous lesions has been demonstrated in several published works. In particular, the use of TNF-alpha inhibitors and IL-1 inhibitors has been described to improve these lesions.

Cytokines produced by T helper cells, including TNF-alpha are described to play an important role in the molecular genesis of BD [69]. Higher soluble TNF-alpha receptors and TNF-alpha sera levels are found in active BD, with their spontaneous secretion by monocytes [70]. This finding supports the possible effective use of anti-TNF-alpha therapy in BD.

#### 2.2.1. Anti-TNF-*α* Inhibitors

In two clinical cases, patients with genital ulcers resistant to azathioprine and prednisone therapy, treated with infliximab (5 mg/kg for a total of 4 infusion) reached a complete remission of the lesion [71, 72]. Hence, infliximab seems to produce good and long-lasting remission of mucocutaneous lesion, after its discontinuation at the 13th infusion as described in two clinical cases in which the patients were treated with infliximab, respectively, in association with azathioprine (0.7 mg/kg/day) and cyclosporine (3 mg/kg/day) [73, 74].

In an open study, the researchers described a good therapeutic response in patients with refractory mucocutaneous lesions, after 12 months of etanercept (50 mg/wk) therapy in addition to conventional immunosuppressive drug (azathioprine and colchicine) with a reduction of oral prednisolone [75]. In a randomized controlled trial (RCT) the effectiveness of etanercept in suppressing most of the mucocutaneous manifestations, such as the oral ulcers, the papulopustular lesions and nodular lesions has been demonstrated, and a lower probability of recurrence of oral ulcers has been described; the genital ulcers do not seem to improve after the treatment [76]. The improvement of genital ulcers after etanercept treatment (25 mg twice week) is described in a case report [77].

#### 2.2.2. Interleukin-1 Inhibitors

Recent studies have explained the role of different chemokines in cellular and molecular pathophysiology of BD [7], and, in particular, interleukin-1 (IL-1) cytokine family is described to play a complex network of minor proinflammatory mediators and subsequent expression of integrins on leukocytes and endothelial cells, with many influences on the inflammatory response [78]. IL-1 has been recently described as a mediator of BD. This innovative concept introduces the identification of new potential targets for biological therapy [6]. Lately, the recombinant human IL-1 receptor antagonist (anakinra), the human immunoglobulin G1 (IgG1) anti-IL-1 beta monoclonal antibody (canakinumab), and the recombinant humanized anti-IL-1 beta antibody (gevokizumab) are proven to be partially useful in the treatment of BD, while appearing to be more effective in ocular involvement [22]. However, with regard to the mucocutaneous lesions, good responses to anakinra have been reported in a patient with oral and genital ulcers resistant to conventional therapy [19] and in a teenager with aphthous lesions and cutaneous lesions unresponsive to traditional treatments [79]. In a case report and in a case series, canakinumab has proved to be successfully used in oral and genital aphthosis, skin lesions, and granuloma annulare [21, 80].

#### 2.2.3. Phosphodiesterase 4 Inhibitors

Finally, apremilast, an inhibitor of phosphodiesterase 4 (PDE), a drug approved for psoriasis and psoriatic arthritis, seems to be a good alternative treatment in BD. In a phase II randomized, placebo-controlled, double-blind study, the apremilast is observed to be effective in the treatment of oral ulcers and in treating genital ulcers [33].

## 3. Conclusions

BD is a complex syndrome, characterized by several clinical manifestations with usually frequent relapses. The mucocutaneous lesions represent nagging and typical manifestations in BD, and their treatment management is usually driven by codominant clinical involvements, such as eyes and gastrointestinal and neurological involvement, or by the subject comorbidity. Although the traditional immunosuppressive drugs, as colchicine, corticosteroids, and azathioprine, are generally effective, several treatment failures, with severe and frequent relapses, troubled the course of the disease. In the last years, new pathogenetic hypothesis supported the use of new biotechnological drugs, as IL-1 inhibitors and TNF*α* inhibitors, in the treatment of BD. Many clinical trials, open studies, and clinical case reports described their efficacy in the treatment of severe mucocutaneous manifestations. Further scientific demonstrations on large scale are necessary to prove their effects on reducing the frequency of mucocutaneous manifestations and on maintaining long-term effects. Several new drugs are under clinical study, but no data are reported yet. A wider drug range, with less adverse events risks, may provide alternative treatment tools for the clinicians, useful in nonresponding patients or in case of adverse events. The main unmet need in the overall management of BD is the lack of a specific treatment to target strategy; therefore, despite several available drugs, the treatment strategy of BD patients is still tailored according to the severity and type of organ involvement. The main goal of therapy in patients with BD is to induce and maintain disease remission and improve quality of life; in this regard, biologics are rapidly becoming effective alternatives to conventional treatments, also in mucocutaneous lesions. Since OA and GU represent relevant threats for quality of life impairment, biological drugs are recommended in mucocutaneous involvement, mainly when traditional drugs miss the mark.

## Figures and Tables

**Table 1:** Brief summary of the main clinical manifestations of Behçet's disease.

Organ involvements	Clinical manifestations	Recommended treatment
Mucocutaneous	Oral aphthae, genital ulcers, pseudofolliculitis, papulopustular lesions, erythema nodosum-like lesions, and pathergy reaction	Colchicine, azathioprine, interferon-*α*, and TNF-*α* antagonist

Eye disease	Recurrent bilateral uveitis (anterior segment, posterior segment, or both), retinal vasculitis, retinal vein occlusion, and optic neuritis	Azathioprine, local or systemic corticosteroids, cyclosporine, infliximab (in combination with azathioprine and corticosteroids), and interferon-*α*.

Gastrointestinal tract	Anorexia, vomiting, dyspepsia, diarrhea, abdominal pain, ulcers, ischemic perforation, thrombosis in the terminal ileum, ileocecal region, and colon	Sulfasalazine, corticosteroids, azathioprine, TNF-*α* antagonista, and thalidomide. In emergency surgical procedures are required such as ileocolectomy or hemicolectomy

Musculoskeletal system	Nonerosive arthritis, nondeforming oligoarthritis, back pain, and sacroiliitis	Colchicine, interferon-*α*, azathioprine, and TNF-*α* antagonists

Cardiovascular system	Vasculitis, superficial thrombophlebitis, deep vein thrombosis, dural sinus thrombosis, occlusion of suprahepatic veins, pericarditis, myocarditis, endocarditis, intracardiac thrombosis, coronary vasculitis, and ventricular aneurysm	Corticosteroids, azathioprine, cyclosporine, and cyclophosphamide

Central nervous system	Severe headache and pyramidal and extrapyramidal symptom (seizures, hemiplegia, and cranial nerve palsies) Central nervous system: focal necrotic cerebral lesions, vascular thrombosis, arterial vasculitis, and aseptic meningoencephalitis Peripheral nervous system: peripheral neuropathies and multiplex mononeuritis	Corticosteroids, interferon-*α*, azathioprine, cyclophosphamide, methotrexate, and TNF-*α* antagonists.

**Table 2:** Overview of works derived from the medical literature reporting treatment indications of mucocutaneous lesions in Behçet's disease.

Drugs	Dose	Authors (year)	Number of patients	Study	Effectiveness
Azathioprine	2,5 mg/kg/day	Yazici et al. (1990) [34]	45	Randomized controlled trial	Oral and genital ulcers and eye involvement

Colchicine	—	Aktulga et al. (1980) [35]	35	Double-blind trial	Erythema nodosum-like lesions
	1-2 mg/day	Yurdakul et al. (2001) [36]	116	Randomized controlled trial	Genital ulcers
	1 mg/day	Davatchi et al. (2009) [37]	169	Randomized controlled trial	Genital and oral ulcers and pseudofolliculitis

Colchicine versus benzathine penicillin + colchicine	—	Çalgüneri et al. (1996) [38]	60/94	Open study	Oral ulcers and erythema nodosum and genital ulcers

Benzathine penicillin versus colchicine versus benzathine penicillin + colchicine	Benzathine penicillin 1.2 million units by injection monthly/colchicine 1 mg day/benzathine penicillin + colchicine	Al-Waiz et al. (2005) [39]	20/21/25	Open study	Oral ulcers and erythema nodosum

Azithromycin		Mumcu et al. (2005) [42]	8	Case series	Folliculitis and oral ulcers
	1500 mg/week for 4 weeks	Mumcu et al. (2013) [43]	10	Case series	Folliculitis and oral ulcers

Minocycline	—	Kaneko et al. (1997) [44]	—	—	—

Thalidomide	100–300 mg/day	Hamuryudan et al. (1998) [45]	98	Randomized controlled trial	Oral and genital ulcers and folliculitis lesions
	50 mg/day	De Wazières et al. (1999) [46]	17	Open study	Oral and genital ulcers
	400 mg/day for 5 days and then 200 mg/day for 4 weeks	Gardner-Medwin et al. (1994) [47]	23	Open study	Oral and genital ulcers
	400 mg/day for 5 days and then 200 mg/day	Saylan and Saltik (1982) [48]	22	Open study	Oral and genital ulcers
	—	Hamza (1986) [49]	—	Open study	—

Cyclosporine	10 mg/kg/day versus colchicine (1 mg/day)	Masuda et al. (1989) [50]	96	Randomized controlled trial	Oral aphthous, dermal lesion, and genital ulcers
	5 mg/kg/day	Assaad-Khalil (1991) [51]	—	Controlled study	Orogenital ulcers and skin lesion
	—	Avci et al. (1997) [52]	—	—	—

Dapsone	100 mg/day	Sharquie et al. (2002) [55]	—	Double-blind placebo-controlled trial	Orogenital ulcers
	—	Sharquie (1984) [54]	—	Open study	—

Interferon alpha 2a	6 million units subcutaneously 3 times per week	Alpsoy et al. (2002) [57]	50	Randomized placebo-controlled and double-blind study	Oral ulcers, genital ulcers, and papulopustular lesion
	9 million units/day three times a week versus colchicine 1,5 mg/day	Boyvat et al. (2000) [58]	20/16	Randomized controlled trial	—
	3 million units subcutaneously daily for 6 months	O'Duffy et al. (1998) [59]	10	Randomized controlled trial	—
	6 million units per day 3 times per week for 2 months	Georgiou et al. (1998) [60]	12	—	Oral aphthae, genital ulcers, erythema nodosum, and pseudofolliculitis
	3 million units/day in the first week (three times a week), 6 million units/day in the second week (three times a week), 9 million units IU/day in the third week and thereafter (three times a week)	Azizlerli et al. (1996) [61]	18	—	Oral aphthae, genital ulcers, erythema nodosum, and pseudofolliculitis
	3 million units three times per week versus 9 million units once a month	Alpsoy et al. (1994) [62]	14	Randomized controlled trial	—
	9 million units three times a week for 6 months	Zouboulis et al. (1993) [64]	10	—	Oral aphthae, genital ulcers, erythema nodosum, pseudofolliculitis
	5 million units 3 times a week for 6 weeks followed by 5 million units once a week for 10 weeks	Hamuryudan et al. (1994) [63]	21	Randomized controlled trial	Oral aphthae, genital ulcers, erythema nodosum, and pseudofolliculitis

Alemtuzumab	—	Perez-Pampin et al. (2013) [67]	1	Case report	Orogenital aphthosis and erythema nodosum
	134 mg	Lockwood et al. (2003) [68]	18	Open study	Orogenital ulcerations

Infliximab	5 mg/kg every 8 weeks	Olivieri et al. (2009) [74]	—	Case report	Mucocutaneous lesions
	5 mg/kg for 4th infusion (0, 2, 6, and 15 weeks)	Haugeberg et al. (2004) [71]	—	Case report	Genital ulcers
	5 mg/kg every 8 + sulfasalazine	Ordahan et al. (2014) [72]	1	Case report	Oral and genital aphthosis
	5 mg/kg every 8 + cyclosporine	Olivieri et al. (2008) [73]	1	Case report	Papulopustulosa, erythema nodosum

Etanercept	50/mg/weekly for 12 months [azatioprine + colchicine]	Mohammed (2014) [75]	15	Open study	Mucocutaneous lesions
	25 mg twice a week	Melikoglu et al. (2005) [76]	40	Randomized controlled trial	Oral ulcers, papulopustular lesions, and modular lesions
	25 mg twice a week	Atzeni et al. (2005) [77]	1	Case report	Genital ulcers

Apremilast	30 mg/twice a day	Hatemi et al. (2013) [33]	55/56	Placebo-controlled double-blind trial	Oral and genital ulcers

Rebamipide	300 mg/day	Matsuda et al. (2003) [56]	14/17	Placebo-controlled double-blind trial	Oral ulcers

Anakinra	100 mg/day	Botsios et al. (2008) [19]	1	Case report	Oral and genital ulcers
	1 mg/kg/day + colchicine	Bilginer et al. (2010) [79]	1	Case report	Oral ulcers and erythema nodosum

Canakinumab	150 mg every 8 weeks	Cantarini et al. (2012) [80]	1	Case report	Recurrent oral and genital aphthosis, erythema nodosum, and pseudofolliculitis
	150 mg every 6 weeks	Vitale et al. (2014) [21]	3	Case series	Mucosal ulcers
